# Ethnobotanical Study of Cultivated Plants in Kaišiadorys District, Lithuania: Possible Trends for New Herbal Based Medicines

**DOI:** 10.1155/2019/3940397

**Published:** 2019-05-26

**Authors:** Zivile Pranskuniene, Kristina Ratkeviciute, Zenona Simaitiene, Andrius Pranskunas, Jurga Bernatoniene

**Affiliations:** ^1^Department of Drug Technology and Social Pharmacy, Lithuanian University of Health Sciences, Kaunas, Lithuania; ^2^Institute of Pharmaceutical Technologies, Lithuanian University of Health Sciences, Kaunas, Lithuania; ^3^The Museum of History of Lithuanian Medicine and Pharmacy, Lithuania; ^4^Department of Intensive Care Medicine, Lithuanian University of Health Sciences, Kaunas, Lithuania

## Abstract

**Background:**

Despite the growing body of ethnobotanical studies in Europe, publications are scarce in Lithuania. Ethnobotanical study in Kaišiadorys district is one of the few from this field done in Lithuania. Lithuania is divided into five ethnographic regions, and Kaišiadorys district is an area which borders with the three of them (Aukštaitija, Dzūkija, and Suvalkija), which determines the ethnographic distinctiveness of the area. The aim of this study was to determine the occurrence of cultivated medicinal plants, their families and pharmaceutical forms used in Kaišiadorys district, Lithuania, and to assess the conformity of medicinal plant materials used by respondents with the recommendations for medicinal plant materials in the World Health Organization monographs.

**Methods:**

The field work was conducted in periods of time from July 2016 to October 2017. During this ethnobotanical research, 30 people were interviewed, 25 of whom agreed to communicate. The average age of respondents was 65 years. Information was collected using semistructured and structured interviews. The obtained information was recorded indicating ethnic names of plants, their preparation techniques, parts used, modes of administration, and application for therapeutic purposes.

**Results:**

Respondents mentioned 71 species of cultivated medicinal plants from 38 families, used for therapeutic purposes and indicated which parts of the plant they use, how they prepare them, indications for use, and ways of administration. The most commonly cited families were Asteraceae (20.5%), Lamiaceae (13.9%), and Apiaceae (12.8%); most popular plants, cited more than 20 times, were* Tilia cordata *L*., Matricaria recutita *L*., Calendula officinalis *L*., Carum carvi *L*.,* and* Artemisia absinthium *L. The frequently used plant parts were flowers (mentioned 35.8%), leaves (16.3%), roots and tubers (16.1%), herb (14.8%), and seeds (7.0%). Diseases most frequently treated were digestive (21.5% of citations), respiratory (21.3 % of citations), mental and behavioral (11.0%) disorders, certain infections and parasitic diseases (10.1%), and diseases of genitourinary system (9.1%).

**Conclusions:**

Only 19 of the cultivated medicinal plant species mentioned by interviewed persons are described in the World Health Organization monographs. This means that the remaining 52 species are used without the World Health Organization approved medical indications, based solely on the folk medicine knowledge and experience. This study showed that the folk use of plants is strongly rooted in daily practice in the studied area.

## 1. Introduction

Next to the modern medicine with new medicines being developed and improved, there remains an interest in traditional ways of folk medicine. People are a part of nature, so naturally, from ancient times, natural resources have been used to keep them healthy or to treat illness. Lithuanian folk medicine, which encompasses various medical knowledge, beliefs, and treatments, existing in the traditional rural community, has deep traditions that are preserved as the nation's cultural heritage [1]. Scientists emphasize the need to integrate folk medicine, or traditional medicine, into the public health system [2]. Research of the knowledge on local medicinal plants and raw materials creates new hypotheses about their impact on human body [3]. The combination of analysis of traditionally used medicinal substances and modern clinical trials may play an important role in the development of new herbal medicinal product formulations based on ethnic heritage [4]. If in the eighties and nineties pharmaceutical companies focused on the synthesis of new medicines, nowadays, the natural medicinal products are being more and more sought for, while using advanced technologies. A new concept for pharmaceutical development was defined in the 1960s: the development of new drugs from natural raw materials is a future perspective; vegetable and animal raw materials could serve as a cheaper “intermediate” product for semisynthetic drugs. Production using plentiful historically approved raw materials would be much more cost-effective for new pharmaceuticals than the full-scale drug synthesis [5].

Medicinal plants are important in social, economic, and cultural terms; however, the traditional knowledge of plant-based materials, especially of less known plants, is constantly diminishing [6]. How often herbal preparations are used depends on their availability and traditions. Scientists are considering how to integrate traditional medical knowledge into the public health system as a complementary treatment tool, effects of which are based on clinical trials [2]. Although modern studies of herbal medicinal preparations are often launched from herbal preparations used in traditional medicine, the new unknown indications for them are still getting discovered [7]. According to the WHO, about 80% of the population in developed countries use products for health care derived from medicinal plants and 30% of all medicines sold in the world contain plant components [8]. In Europe, over 1,300 local plants are recognized in the medicine [9].

In Lithuania, the history of using plant-based materials is rich and can be analyzed geographically, culturally, and linguistically. Medicinal plants have been collected or grown for a long time. Also various spices, imported vegetable raw materials, and knowledge of their use have become available through trade routes [10]. Scientists believe that traditional Lithuanian medicine uses 462 spontaneous, adventive, or introduced higher plant species, five fungi, two lichens, one moss, and one alga species [11]. Since old times in Lithuania, plants grown in flower beds had not only decorative purposes. Most types of ornamental plants traditionally grown in Lithuania were used for treatment. Farmstead landscaping and the appearing of flower beds have been influenced by the Valakas land reform performed in the Grand Duchy of Lithuania and by the establishment of monasteries. Fruit and vegetable gardens and flower beds were built at the monasteries, flowers, vegetables, and shrubs and fruit trees were grown there. For example, the pharmacy of Pažaislis Monastery (founded in 1662) was like a laboratory where monks worked with medicinal plants, studied medical literature, manuals and pharmacopoeia, and created recipes. The monasteries' recipes abounded in both local and imported medicinal raw materials [12]. Until the beginning of the 20^th^ century, imported raw materials used to reach Europe through London, Amsterdam, or Hamburg from Java, e.g.,* Cort. Chinae*, South America, e.g.,* Lign. Guajaci, Rad. Rhei, Flor. Chamomillae, Aloe, *and* Sanguis Draconis* [10]. Jurgis Ambraziejus Pabrėža the vicar of the Kretinga Monastery, the first botanist of the 19^th^ century Western Lithuania, and the author of the first Lithuanian botanical terminology work (J. Pabrėža manuscript “Taislius augyminis” (Remedies of Plant Origin)) was included in the UNESCO World Memory Program in 2008. In the Kretinga monastery he cultivated about 400 plant species in plots. J. A. Pabrėža classified plants by their purpose: (1) those used for decorations, (2) those used in medicines, (3) those used by farmers and craftsmen, and (4) dye plants [13]. Modern ethnopharmaceutical research in Europe is growing rapidly, especially in Southern European countries, Spain, and Italy. Now it focuses on “unnoticed” Eastern European countries, Poland, Romania, Bulgaria, and Lithuania [14]. Despite the increasing number of ethnopharmaceutical studies in Europe, the number of publications from Lithuania in scientific journals is minimal [15–17]. Several previous studies have shown that it is important to collect and systematize this information as quickly as possible, to preserve it as a part of traditional Lithuanian heritage and to successfully use it in further research.

Ethnobotanical study in Kaišiadorys region is one of the few from this field done in Lithuania. Lithuania is divided into five ethnographic regions, among them Kaišiadorys district borders with Aukštaitija, Dzūkija, and Suvalkija. This fact determines the ethnographic distinctiveness of the region. Scientists in Lithuania seek to create unique herbal medicinal products from traditionally used plants, the use of which has been approved historically, taking into account the needs of today's consumer.

Tasks of the work are to determine the distribution of cultivated medicinal plants in Kaišiadorys district, Lithuania, their families and pharmaceutical forms, to perform the analysis of indications for the use of medicinal plants and to identify the types of cultivated medicinal plants that had been used / still are used for treatment in Lithuanian traditional medicine and are possibly suitable for the formulation of herbal medicinal preparations based on ethnic heritage, and to assess the conformity of plant materials used by the respondents with recommendations in the World Health Organization monographs.

## 2. Material and Methods

### 2.1. Study Area

The survey was carried out in Kaišiadorys district, located in the middle part of Lithuania, Kaunas region, in Kaunas Lagoon, the rivers Nemunas and Neris. This area is interesting because it has intertwined dialects, cultural, architectural, and art traditions, and customs of three Lithuanian ethnographic regions. Theoretically, Kaišiadorys district belongs to Aukštaitija ethnographic region, but it also borders with Dzūkija and Suvalkija [18]. The different traits of the ethnographic regions and their traditions determine the peculiarities of traditional folk medicine in these different areas.

Kaišiadorys is a town located 67 km west of Vilnius and 37 km east of Kaunas [19]. The name of the town was first mentioned in 1590 under name of Košeidarova being mentioned. In 1902, Kaišiadorys settlement became a town. The growth of the town was determined by the large flows of people traveling by railway, passed through Kaišiadorys and transferred to another railway line, which led to the establishment of the main businesses: shops, hotels, and inns, the majority of the population being Jews [19]. In earlier times the majority of the residents of Kaišiadorys and the surrounding towns were Jews, and although their traditional folk medicine would also be a valuable subject for research, Jewish respondents were not recorded in this study.

Kaišiadorys became the district center in 1950 [19]. The area of the district is 1087 km², which makes 1.7% of the total area of Lithuania. According to the data of 2015, 31 624 inhabitants lived in the district.

There are two towns in the district, Kaišiadorys and Žiežmariai, three settlements, Kruonis, Rumšiškės, and Žasliai, and 384 villages [20].

**Figure 1:**
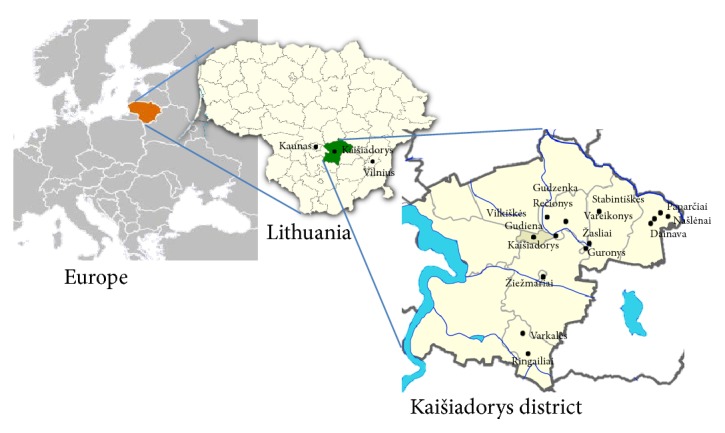
Study area.

Respondents living in Kaišiadorys and Žiežmariai and in the 12 villages of the district were interviewed during the research.  Figure 1 shows the territory of Kaišiadorys district with the marked areas of survey.

63% of the Kaišiadorys district population lives in villages [20]. The majority of the survey respondents also were rural residents: 28% of the respondents lived in the urban area but came from rural areas, while the remaining 72% lived in villages.

The diversity and occurrence of plants in both the country and the region are determined by geographical location and climatic conditions. The territory of Lithuania is in the mid-latitude climate zone; only the Baltic coastal climate area is closer to the Western European climate [21]. The standard climate normal in Lithuania (1981-2010) shows 18°C being the highest and -3°C being the lowest monthly average temperature [22]. The maximum average monthly precipitation based on the standard climate normal (1981-2010) is about 75-80 mm and the lowest is about 35-40 mm [23].

In the north-western part of Kaišiadorys district territory there is a lowland of the lower reaches of Neris, in the southeast of the Dzūkai heights, characterized by ash forests with hornbeams, and the Aukštadvaris forest. The average temperature of January is –5.1°C and July + 17.5°C. In Kaišiadorys district, 631 mm of precipitation falls annually. There are 39 lakes and eight ponds in the district. The forest area of Kaišiadorys district is 31.1%. 63% of all trees are coniferous: 38% pine and 25% spruce. In mixed forests, usually grow broad-leaved trees and spruce trees. The largest swamp is Palaraistis. The territory of Kaišiadorys district includes parts of Kaunas Lagoon and Aukštadvaris regional parks and a few reserves: Lapainis botanical, Būda and Kaukinė botanical-zoological, Lomena, Budeliai, and Strošiūnai landscape, and Gabriliava pedological reserve [20].

### 2.2. Methods

The purpose of the study was explained to each interviewed person, prior informed consent was obtained from all interviewees, and all conversations were recorded and encoded following the Code of Ethics of the International Society of Ethnobiology [24]. The research was approved by the Bioethics Center of Lithuanian University of Health Sciences (No. BEC-FF-18). Approval to carry out this type of research was obtained from the authorities of local community. Field work was conducted in periods from July 2016 to October 2017. During this ethnobotanical research, 30 people were interviewed, 25 of whom agreed to communicate, and 24 of them were females and 1 male. As we have mentioned in another study done in Lithuania, the main reason for higher number of women was the fact that traditional knowledge of herbal medicine in Lithuania was passed down through the female line [17]. The majority of respondents in the study (64%) were of older generation, 24% of whom were aging adults (60-74 years) and 40% were elderly (75-90 years). Middle-aged (30-59 years) people made up 36% of the respondents. Many of our respondents lived in lonely farmsteads with the nearest farmstead or settlement a few kilometers away. Information was collected using semistructured and structured interviews. Respondent search used guides and random selection and snow ball techniques. In order to reduce the number of random respondents and to form a target group more efficiently, in the search of respondents, guides who knew the respondents and their possible experience in traditional folk medicine and in herbal medicine were used. The guides of our study were two women: a resident of Paparčiai village, the chairman of the community and the employee of the culture center, and a long-time head of Ringailiai folklore team, who also worked in the culture center. Their work specifics provided them with an opportunity to get to know the people of the country and to make contacts with them. A field study conducted with a guide is rewarded by the favor and openness of the respondents and is likely to capture more ethnopharmaceutical information during a structured interview. At the end of each interview, the respondents were asked whether they knew more people who were engaged in herbal medicine or were interested in traditional folk medicine; thus the target group was increased applying the snowball method, where one respondent mentions another potential respondent.

The first meeting with the interviewed persons occurred at their homes or gardens. During interviews, the investigator used a complete questionnaire and wrote down himself the answers to the questions. The obtained information was recorded indicating ethnic names of plants, their preparation techniques, parts used, modes of administration, and application for therapeutic purposes. Much attention was paid to the sources of ethnobotanical knowledge obtained by the respondents.

In order to get more information which the respondents naturally missed during the first meeting, some respondents were met more than once. Parts of plants were identified using writings on traditional Lithuanian flora [25–27]. Also, when possible, the respondents were asked to supply a fresh sample of each plant mentioned for the taxonomic determination and name it in a local dialect. Taxonomic identification, botanical nomenclature, and family assignments followed the Plant List database [28] and the Angiosperm Phylogeny Group IV [29]. The frequency of reports indicates the number of respondents who mentioned the use of each species (Table 1).

## 3. Results and Discussion

### 3.1. Demographic Data of the Respondents and Sources of Knowledge

In the ethnobotanical survey in Kaišiadorys district, 25 respondents, 90% of whom lived in villages, were interviewed in the structured interviews. The authenticity of ethnopharmaceutical knowledge of Kaišiadorys district could have been influenced by the previous place of residence of the respondents, as it might be based on the knowledge from there, but majority of the respondents lived in Kaišiadorys district, and only 16% of them lived previously in another area. Taking into account the fact that the older rural inhabitants tend to preserve old traditions of their country and community, it can be expected to have obtained pure ethnobotanical knowledge of Kaišiadorys district. The average age of respondents was 65 years.

The higher number of older generation respondents allowed expecting obtaining genuine local ethnobotanical knowledge passed down the generations, rather than being based on the information presented by modern media. The education, profession, and sources of folk medicinal knowledge may also influence the authenticity of ethnopharmaceutical knowledge. All older respondents (40%) had completed grades 3, 4, or 7, which is an incomplete secondary education, while the remaining elderly and middle-aged respondents had completed secondary education (28%), upper secondary education (16%), or higher education (4%).

Respondents, who had not completed their secondary education, when talking about the profession, mentioned working in collective farm, singing in church, community-specific rituals, and making crafts; those with higher education mentioned professions such as folklore team leader, ceramicist, ethnocultural teacher, nurse assistant, and nurse.

**Figure 2:**
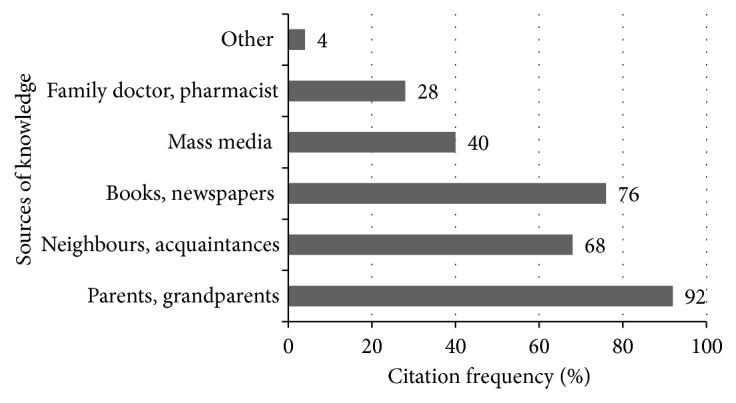
Respondents' sources of ethnobotanical knowledge.

Most of the respondents did not have higher education and had not even acquired secondary education, and the mentioned professions, such as collective farm work, head of folklore team, making crafts, or ethnocultura teacher, suggested that respondents were closely related to folk traditions; therefore, the ethnobotanical knowledge obtained during the research is based on the knowledge of traditional folk medicine of Kaišiadorys district, passed and saved from generation to generation. Respondents provided sources of ethnobotanical information, from which they gathered knowledge about the use of medicinal plants for the treatment of various disorders. The sources of ethnopharmaceutical knowledge of the respondents are presented in Figure 2.

Most respondents learned to treat with herbs from their parents, grandparents (92.0% of citations), and from their neighbors or acquaintances (68.0% of citations), which indicates the existence of an oral tradition when information is passed by word of mouth. However, not any less important source of ethnobotanical knowledge were books and newspapers (76.0% of citations), readily available nowadays and useful for both remembering forgotten information and supplementing existing knowledge. A smaller part of the material collected in the study was based on the knowledge from the mass media (40.0% of citations), which was less popular among the older generation. Respondents also relied on information provided by a family doctor or pharmacist (28.0% of citations) and other sources (4.0% of citations) identified as knowledge derived from sorcerers.

In order to preserve the knowledge of traditional folk medicine, it is important to write it down or pass it on to others. 64% of respondents said that they passed on their knowledge to other people, who were usually family members or close acquaintances, neighbours, or friends. Most often older respondents mentioned that they told their grandchildren about medicinal plants or shared the knowledge with their peers and neighbours.

### 3.2. Medicinal Plants Cultivated for Medicinal and Other Purposes

The primary function of home gardens generally is food production but it also serves other purposes, such as medicinal and ornamental ones. Plant cultivation at home has many advantages: for better harvests plant species are selected, land prepared and proper conditions for them are chosen, and plants are always “at hand” [14]. The studies have distinguished the same plants used for various purposes: food (*Chamaenerion angustifolium *L*., Heracleum lanatum *Michx., etc.), medicines (*Heracleum lanatum*) or for household use (*Juniperus sibirica* Burgsd.) and for incense (*Leymus mollis *(Trin.) Hara), and* Urtica *sp. L. for fiber production, as well as data on local peoples' perception of the peculiarities and differences of plant cultivation [30]. The use of vegetable raw materials in households is also linked to their specific purposes [31].

Knowledge about cultivated plants and their use can be found in the material of the first Lithuanian ethnographic expeditions [32]. Ethnopharmaceutical research on ornamental plants in Lithuania has just started. There have been described 153 species of ornamental plants, including 17 trees, 13 shrubs, 123 herbaceous plants, and 13 species at home as indoor plants. 125 of the total number of studied plants were used for medical purposes (81.7%). The most abundant species belong to Asteraceae, Lamiaceae, Rosaceae, Solanaceae, and Fabaceae families [33]. Comparing therapeutic indications in Lithuanian folk medicine with the 19^th^ century's and the modern medicine, most coincidence was found in the use of chamomile (*Matricaria recutita* L.), calendula (*Calendula officinalis *L.), absinth wormwood (*Artemisia absinthium *L.), horseheal (*Inula helenium* L.), and juniper. The most frequently used parts were aerial parts, leaves, and flowers, from which aqueous and ethanol extracts were commonly prepared [33].

Historically, in families women had more to do with plant species. In most societies cooking, healing with traditional medicines, dyeing cloth and yarns, gardening, and seed selection belong to female activities [34]. In Lithuania, the trend was the same [35]. It can be assumed that there were medicinal raw materials possible to find near the house in illness.

We registered 71 species of medicinal plants from 38 families used for therapeutic purposes. The results of the research are presented in Table 1. The most commonly cited families were Asteraceae (20.5%), Lamiaceae (13.9 %), and Apiaceae (12.8%). The most popular plants cited more than 20 times were* Tilia cordata *Mill.,* Matricaria recutita, Calendula officinalis, Carum carvi *L., and* Artemisia absinthium*. The most commonly used plant for prophylaxis was* Tilia cordata* mentioned 25 times. Respondents pointed out that they usually use flowers to make tea for colds, fever, or cough, symptoms of flu, in order to promote sweating. One respondent also indicated that the flowers are used to prepare inhalations for respiratory diseases and buds to chew for increased gastric acidity. The second place is taken by chamomile (*Matricaria recutita*, cited 24 times). The herbal tea is prepared from its flowers for gastrointestinal disorders such as inflammation, pain, bloating, and diarrhea, less commonly for colds or coughs. One respondent emphasized that chamomile tea is given to breastfed babies to avoid abdominal colic. Respondents also pointed out that decoctions of both flowers and aerial parts can be used to wash eyes and infected wounds, applied as poultices in skin inflammation, to bath children, so that they are calm, and to rinse inflamed oral cavity or throat. Raspberry (*Rubus idaeus* L.) and calendula (*Calendula officinalis*) were mentioned 22 times. Raspberry stems and leaves are used to prepare tea for colds, fever, or cough. Respondents mentioned that they use marigold flowers for tea, decoction, or ointment. Tea is taken for mouth, throat, stomach, intestine, urinary tract, and uterus inflammation and in menstruation. The decoction is used for mouth or throat rinses, washes, or baths in menstruation and for treatment of vaginal and urinary tract inflammation. Marigold flowers ointment is used to treat skin scars. Ethnobotanical studies carried out in various European countries have found that terrestrial parts of medicinal plants are commonly used for treatment or prophylaxis [36–38]. The respondents mentioned that they do not use all parts of the plant together, but a different part in each case, knowing that those particular parts of the plant contain needed active ingredients and seeking to have the best effect in treating the respective ailments or diseases. Parts of plants the most used for treatment were flowers (mentioned 35.8%), leaves (16.3%), roots and tubers (16.1%), aerial parts (14.8%), and seeds (7.0%). The most popular modes of preparation were tea (47.5% of citations), raw material (23.4%), poultice (7.4%), and decoction (6.7%). According to the respondents' experience, tea is easily and quickly prepared by pouring hot water over raw materials and keeping for 5-10 minutes. The second way of use mentioned by respondents is a raw material or functional food. The popularity of this method of preparation may be due to the fact that it does not require much time or effort and is simple. It includes the use of fresh raw materials, for example, eating strawberries, wild strawberries, beetroot, or cabbage leaves, as well as raw material prepared as food. Throughout the world and Europe, the research has found that medicinal herbal raw materials were used for therapeutic purposes as well in the form of ready-to-eat vegetables, jams, or spices [39–41]. Talking about poultices, there were mentioned ways of their preparation such as putting fresh raw material directly on the affected parts of the body or using gauze soaked with infusions, extracts, or decoctions. Preparation of decoctions takes longer time, which is likely to have led to its less frequent use in the preparation of herbal raw materials. Other ways of preparing and using medicinal herbal raw materials include ointments, sprinkles, cushions, bath brooms, oil infusions, oil extraction, and growing plants indoors.

### 3.3. Indications for the Use and Comparison of Indications with the World Health Organization Monographs

**Figure 3:**
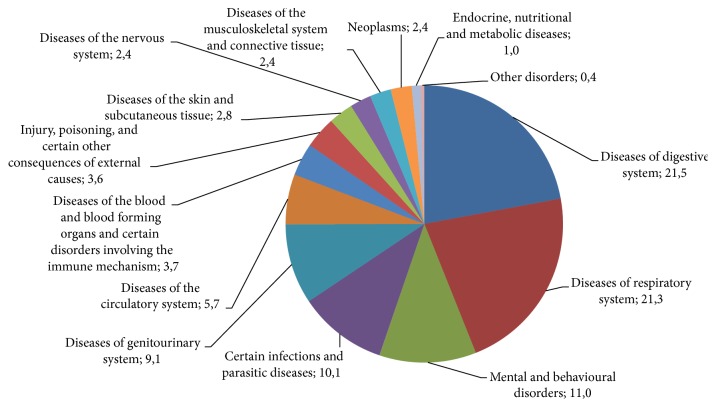
Indications of use by respondents of medicinal herbal raw materials (data are presented in %).

Once the obtained information is structured in accordance with the system of International Classification of Diseases [42], it can be observed which diseases, disorders, or ailments are most commonly treated by respondents with collected herbal material. Data on the indications of use by respondents of medicinal herbal raw materials are presented in Figure 3. Medicinal plants were most frequently used for the treatment of digestive (21.5%) and respiratory (21.3%) diseases, mental and behavioral disorders (11.0%), certain infections and parasitic diseases (10.1%), and diseases of genitourinary system (9.1%).

Gastrointestinal disorders most often mentioned by respondents were diarrhea, intestinal gas accumulation, gastric mucosal lesions, ulcers, digestive, and liver or gall bladder disorders. Respiratory ailments commonly referred to were pneumonia, flu, colds, bronchitis, asthma, or symptoms such as cough, sore throat, inflammation, and swelling of the nasal mucosa during a cold. As urinary system disorders, there were mentioned kidney disorders, kidney stones, urinary tract infections, and diuresis. Gender-related ailments include menstrual disorders such as pain, excessive bleeding, or total absence of secretions. Mental and behavioral disorders commonly referred to were anxiety, insomnia, and depression or fright.

According to the World Health Organization, the use of herbal raw materials for therapeutic purposes in developing countries has increased in recent decades [43, 44]. Although only a small proportion of medicinal plants have scientifically based indications for use, information on the safety and efficacy of medicinal herbs is important for both the average consumer and the healthcare professional. Therefore, one of the objectives of the World Health Organization's ongoing activities is to disseminate scientific information on folk medicine to ensure the safety and quality of medicinal products used in folk medicine [45]. According to the World Health Organization (WHO), there are currently few plant species that are scientifically tested and evaluated based on their medical application. There are even less plants that have been approved for safe and effective use for medical purposes. A guarantee of the safety, efficacy, and quality of medicinal plants in developed countries is challenging as people increasingly return to herbal remedies refusing to use chemical drugs [43]. According to the WHO recommendations on safety and efficacy, the use of medicinal herbal raw materials in Kaišiadorys district was compared with the recommended use of medicinal herbs in the WHO monographs.

Respondents in Kaišiadorys region mentioned 71 species of cultivated medicinal plants from 38 families used for therapeutic purposes. Only 19 of them are described in the WHO monographs.

While most of the indications for the use of medicinal herbal raw materials recorded in the study match the recommendations in the WHO monographs, some discrepancies have been observed; for example, the respondents used flowers of* Tilia cordata* for cold and cough as stated in the WHO monographs, but they used its buds as well, which were chewed in the presence of increased gastric acidity.

The WHO monographs have confirmed the external use of* Calendula officinalis*; however, respondents as well used the flower tea internally to treat inflammations of the throat, stomach, intestine, uterus, and urinary tract.

Indications for the use of common dill (*Anethum graveolens* L.) totally differed: the WHO monographs recommend the intake of fruit in the treatment of dyspepsia, gastritis, gas accumulation, and stomach pain, while the respondents used seeds, flowers, leaves, and herb internally for high blood pressure. Respondents not only used peppermint (*Mentha x piperita* L.) leaves internally, as indicated by the WHO, but also used the whole herb externally in acne.

The discrepancy between indications of use of medicinal raw herbal materials recorded in Kaišiadorys district and the WHO monographs shows that the information provided by respondents is not based on scientific literature or research, but on the long-standing experience of traditional folk medicine, which is passed down from generation to generation.

## 4. Conclusions

Only 19 of cultivated medicinal plant species mentioned by interviewed persons are described in the World Health Organization monographs. This means that the remaining 52 species were used without World Health Organization approved medical indications and were based solely on the folk medicine knowledge and experience. The study showed that the folk use of plants in the studied area was strongly rooted in daily practice. In Lithuania, researchers use traditional plants according to their historical background and try to create unique products suitable for today's user needs.

The most suitable for Lithuanian climatic conditions cultivated plants with historically justified medical applications can be a source of ideas for further research.

## Figures and Tables

**Table 1:** Cultivated herbal materials in Kaišiadorys district.

*Family*	*Botanical name*	*Disorder* (frequency of citations)	*Part used*	Preparation	*Administration and usage* (O. Ad.: oral administration, Ext.: external use)	*Other purposes* (spice: C, decorative: D, and food: F)
Acoraceae	*Acorus calamus* L.	Gastric disorders (1)	Roots	Raw material	O. Ad.	D
		Pain of gum (1)	Roots	Compress	Ext.	
			Roots	Decoction	Ext., rinsing	

Alliaceae	*Allium cepa* L.	Weak organism (8), helminth (2)	Corm	Raw material	O. Ad.	C F
		Wounds (1)	Corm	Compress	Ext.	

Alliaceae	*Allium sativum* L.	Hypertension (3)	Corm	Raw material	O. Ad.	C F
		Helminth (9)	Corm	Raw material	O. Ad.	
			Roots	Decoction with milk	O. Ad.	
		Weak organism (7)	Corm	Raw material	O. Ad.	
		Inflammation of the throat (2)	Corm	Raw material	O. Ad.	
		Inflammation of the mucous membrane of the nose (1)	Corm	Raw material	O. Ad.	
		Oncology diseases (1)	Corm	Raw material	O. Ad.	
		Toothache (1)	Corm	Chewing or compress	Ext.	

Aloaceae	*Aloe arborescens* Mill.	Gastric wounds (2)	Leaves	Chopped leaves are mixed with ethanol and honey	O. Ad.	D
		Lung diseases (2), cough (2)	Leaves	Chopped leaves are mixed with honey	O. Ad.	
		Weak organism (1)	Leaves	Chopped leaves are mixed with honey and lemon juice	O. Ad.	
		Sore throat (1)	Leaves	3 chopped leaves are mixed with 3 chopped lemons, 12 quail eggs, and honey	O. Ad.	
		Skin wounds, burns (2)	Leaves	Juice	Ext.	
		Herpes (1)	Leaves	Juice	Ext.	

Apiaceae	*Anethum graveolens* L.	Hypertension (16)	Seeds, flowers, leaves, and aerial parts	Tea	O. Ad.	C F

Apiaceae	*Carum carvi* L.	Abdominal bloating (15)	Seeds	Tea	O. Ad.	C F
		Indigestion (8)	Seeds	Tea	O. Ad.	
		Diarrhea (3)	Seeds	Tea	O. Ad.	
		Headache caused by fatigue and stress (1)	Seeds, flowers	Tea	O. Ad.	
		Lactation disorder (1)	Seeds	Tea	O. Ad.	

Apiaceae	*Daucus carota* L.	Gastric hyperacidity (1)	Roots	Raw material	O. Ad.	F
		Diarrhea (1)	Roots	Porridge	O. Ad., eating of porridge all day	
		Helminth (2)	Roots	Raw material	O. Ad.	
		Weak vision (1)	Roots	Raw material	O. Ad.	
		Tonisation (1)	Roots	Tea	O. Ad.	

Apiaceae	*Petroselinum crispum* Mill.	Inflammation of the bladder (5)	Aerial parts	Tea	O. Ad.	C F

Asteraceae	*Artemisia absinthium* L.	Indigestion (9), appetite (8), biliary disorders (2), diarrhea (6), and abdominal, gastric pain (6)	Flowers	Tea	O. Ad.	D
		Helminth (2)	Flowers	Tea	O. Ad.	
		Detoxification of the organism (1)	Flowers	Tea	O. Ad.	
		Insomnia (1)	Flowers	Tea	O. Ad.	

Asteraceae	*Calendula officinalis* L.	Inflammation (of the mouth, throat, stomach, intestine, and urinary tract) (19), painful menstruations (8)	Flowers	Tea	O. Ad.	D
		Inflammation of the mouth, throat (4)		Decoction	Ext., rinsing	
		Menstruations (10), inflammation of the urinary tract, vagina (6)		Decoction	Ext., wash of genital organs, bath	
		Wounds (1)	Flowers	Decoction	Ext., wash of wounds	
		Skin scars (1)	Flowers	Ointment	Ext.	

Asteraceae	*Echinacea purpurea* L. Moench	Weak organism (2)	Flowers	Tea	O. Ad.	D

Asteraceae	*Helianthus annuus* L.	Outgrowths (1)	Roots	Decoction	O. Ad.	D F

Asteraceae	*Inula helenium* L.	Eczema (1), bone cracks (1)	Roots	Ointment: chopped roots are heated with fats	Ext.	D

Asteraceae	*Matricaria recutita* L.	Gastric and intestinal disorders (12)	Flowers	Tea	O. Ad.	D C
		Abdominal bloating of infant (1)	Flowers	Tea	O. Ad.	
		Cold (2), cough (3), and runny nose (3)	Flowers	Tea	O. Ad.	
		Inflammation of the throat (3), mouth (3)	Flowers	Decoction	Ext., rinsing	
		Inflammation of the eyes (4), wounds (6)	Flowers, aerial parts	Decoction	Ext., wash	
		Inflammation of the skin (1)	Flowers, aerial parts	Decoction	Ext., compress	
		Calming effect on children (1)	Flowers, aerial parts	Decoction	Ext., bath	

Asteraceae	*Silybum marianum* L. Gaertn.	Hepatic disorder (1)	Flowers	Tea	O. Ad.	D
		Detoxification of the organism (1)	Leaves	Tea	O. Ad.	

Asteraceae	*Tanacetum balsamita* L.	Being used for oral hygiene (1)	Aerial parts	Chewing	Ext.	D

Berberidaceae	*Berberis vulgaris* L.	Hepatic inflammation (1)	Roots	Decoction	O. Ad.	D

Betulaceae	*Betula pendula* Roth.	Blood thrombus (5)	Buds	Tea	O. Ad.	D
		Cough (1)	Buds	Tea	O. Ad.	
		Weak organism (1)	Buds	Tea	O. Ad.	

Boraginaceae	*Symphytum officinale* L.	Bone cracks (4), contusion (3), joint (2), muscle (1) pain, and sprain (1)	Roots	Ethanol infusion, chopped roots are mixed with butter or unsalted fats, compress	Ext., compress, rubbing	D
		Gastric ulcer (1)	Roots	Ethanol infusion	O. Ad.	

Brassicaceae	*Armoracia rusticana* G. Gaertn., B. Mey. & Scherb.	Headache (1)	Leaves	Compress	Ext.	F C

Brassicaceae	*Brassica oleracea* L. var. *capitata*	Colon cancer (1)	Leaves	Raw material	O. Ad.	F
		Blood clotting disorders (1)	Leaves	Raw material	O. Ad.	
		Constipation (1)	Leaves	Pickled	O. Ad.	
		Swelling (4), inflammation (4), and headache (4)	Leaves	Compress	Ext.	

Cannabaceae	*Humulus lupulus* L.	Anxiety (4)	Buds	Tea	O. Ad.	D
			Buds	Pillow	Ext.	

Chenopodiaceae	*Beta vulgaris* L. subsp. *vulgaris*	Anemia (3), lack of iron (3), increased blood clotting (3)	Roots	Raw material, juices	O. Ad.	F
		Weak organism (1)	Roots	Raw material, juices	O. Ad.	

Commelinaceae	*Callisia fragrans* (Lindl.) Woodson	Joint pain (1)	Leaves: it should be 9 parts of stem; then leaves are appropriate for use	40-degree ethanol infusion for compress	Ext.	
		Weak organism (1)	Leaves	Ethanol extract	O. Ad.	

Convallariaceae	*Convallaria majalis* L.	Contusion, swelling (1)	Aerial parts	Compress of water extract	Ext.	D

Cupressaceae	*Juniperus communis* L.	Outgrowths (1)	Roots	Ethanol infusion	O. Ad.	D C F
		Blood detoxification (2)	Roots	Water infusion	O. Ad.	

Elaeagnaceae	*Hippophae rhamnoides* L.	Weak organism (1)	Fruits	Juice	O. Ad.	F D
		Gastric ulcer (1)	Fruits	Oil	O. Ad.	

Fabaceae	*Phaseolus vulgaris* L.	Hypertension (1)	Seeds	Eating of boiled or roasted seeds	O. Ad.	F D

Fabaceae	*Trifolium pratense* L.	Male potency disorders (2)	Flowers	Tea	O. Ad.	D
		Headache during menstruation, menopause (3)	Flowers	Tea	O. Ad.	

Fabaceae	*Trifolium repens* L.	Cold (2)	Flowers	Tea	O. Ad.	D

Fagaceae	*Quercus robur* L.	Diarrhea (4)	Bark	Decoction	O. Ad.	D F

Geraniaceae	*Pelargonium odoratissimum* (L.) L'Her.	Cold (2), inflammation (2)	Flowers, leaves	Tea	O. Ad.	D
		Being used for air disinfection (1)	Aerial parts	Fumigation	Ext.	

Grossulariaceae	*Ribes nigrum* L.	Cold (7), fever (3), and cough (1)	Leaves, fruits, and stem	Tea	O. Ad.	F D
		Weak organism (3)	Leaves, fruits, and stem	Tea, eating of fruits	O. Ad.	
		Blood clotting disorders (1)	Leaves, fruits, and stem	Tea	O. Ad.	
		Insomnia, anxiety (2)	Leaves	Tea	O. Ad.	
		Being used for regulation of glucose for diabetics (1)	Leaves, fruits, and stem	Tea	O. Ad.	

Grossulariaceae	*Ribes uva-crispa* L.	Renal disorders (1)	Flowers	Tea	O. Ad.	D F

Hippocastanaceae	*Aesculus hippocastanum* L.	Vein disease (1)	Flowers	Ethanol infusion	Ext., rubbing	D

Lamiaceae	*Agastache foeniculum* (Pursh) Kuntze	Depression (4)	Leaves, flowers	Tea	O. Ad.	D F C
			Leaves, flowers	Pillow	Ext.	
		Cold (1)	Leaves, flowers	Tea	O. Ad.	
		Hypertension (1)	Leaves, flowers	Tea	O. Ad.	

Lamiaceae	*Elsholtzia ciliata* (Thunb.) Hyl.	Appetite disorders (1)	Flowers	Tea	O. Ad.	D C
		Diuresis disorders (1)	Flowers	Tea	O. Ad.	

Lamiaceae	*Melissa officinalis* L.	Anxiety, insomnia (14)	Aerial parts, leaves	Tea	O. Ad.	D C
		Diuresis and release of intestinal gas disorders (1)	Aerial parts	Tea	O. Ad.	
		Appetite disorders (1)	Aerial parts	Tea	O. Ad.	

Lamiaceae	*Mentha x piperita* L.	Anxiety (14), insomnia (14)	Leaves, aerial parts	Tea	O. Ad.	D C F
		Indigestion (6), diarrhea (1), and meteorism (1)	Leaves, aerial parts	Tea	O. Ad.	
		Acne (1)	Aerial parts	Compress of strong tea	Ext.	

Lamiaceae	*Nepeta cataria* L.	Headache (1)	Aerial parts	Tea	O. Ad.	D

Lamiaceae	*Origanum vulgare* L.	Appetite disorders (1), indigestion (3)	Aerial parts	Tea	O. Ad.	D C F

Lamiaceae	*Salvia officinalis* L.	Sore throat (4)	Leaves	Decoction	Ext., rinsing of the throat	D
			Leaves	Tea	O. Ad.	
		Being used for strengthening of voice strings (1)	Leaves	Tea	O. Ad.	
		Mental illness (1), tremor of hands (1), fright (1), and stress (1)	Leaves	Tea	O. Ad.	

Linaceae	*Linum usitatissimum* L.	Gastric, esophageal inflammation (1)	Seeds	Decoction	O. Ad.	F D

Malvaceae	*Althaea officinalis* L.	Cough (1)	Roots	Tea	O. Ad.	D

Malvaceae	*Malva neglecta* Wallr.	Gastric hyperacidity (1)	Flowers	Raw material	O. Ad.	D

Oleaceae	*Syringa vulgaris* L.	Wounds (1)	Leaves	Compress	Ext.	D
		Nervousness (1)	White flowers	Tea	O. Ad.	

Onagraceae	*Chamaenerion angustifolium* L.	Gastric wounds (1), intestinal diseases (1)	Leaves	Infusion	O. Ad.	D

Paeoniaceae	*Paeonia sp. *L.	Fright (4)	Flower: pink flowers for girls and white flowers for boys (2)	Tea	O. Ad.	D

Pinaceae	*Pinus sylvestris* L.	Tuberculosis, (1), lung inflammation	Bud	Buds boiled with milk	O. Ad.	D
		Respiratory tract diseases (5), bronchitis, (5), and cough (5)	Cone	Ethanol infusion	O. Ad.	
			Bud	Ethanol extract, ethanol infusion, tea, mixed with honey, mixed with sugar	O. Ad.	
			Bud	Bath, inhalation	Ext.	
		Gastric wounds (1)	Bud	Tea	O. Ad.	

Poaceae	*Avena sativa* L.	Lungs inflammation (1)	Seeds	Compress	Ext.	F

Poaceae	*Secale cereale* L.	Erysipelas (1)	Seeds	Powders	Ext.	

Primulaceae	*Primula veris* L.	Inflammation of the bladder (1)	Aerial parts	Tea	O. Ad.	D
		Cold (2)	Aerial parts	Tea	O. Ad.	

Rosaceae	*Agrimonia eupatoria* L.	Sore throat (1)	Aerial parts	Infusion	Ext., rinsing of the throat	

Rosaceae	*Aronia melanocarpa* (Michx.) Elliott.	Blood clotting disorders (1), lack of iron (1)	Fruits	Raw material	O. Ad.	D F
			Fruits	Tea	O. Ad.	

Rosaceae	*Crataegus monogyna* Jacq.	Cardiac disorders (4)	Fruits	Raw material	O. Ad.	D
			Flowers, bark	Ethanol infusion	O. Ad., used at the morning before eating	

Rosaceae	*Fragaria *sp. L.	Decreased hemoglobin in the blood (1)	Fruits	Raw material	O. Ad.	D F

Rosaceae	*Padus avium* Mill.	Erysipelas (2)	Flowers	Tea	O. Ad.	D
			Flowers	Bath, wash	Ext.	

Rosaceae	*Prunus cerasus* L.	Fever (1)	Stem	Tea	O. Ad.	D F

Rosaceae	*Rosa canina* L.	Weak organism (1)	Fruits	Tea	O. Ad.	D F

Rosaceae	*Rubus rosifolius* Sm.	Being used as vitamin source for diabetics (1)	Fruits	Raw material	O. Ad.	D F

Rosaceae	*Sorbus aucuparia* L.	Wounds (1)	Bark	Decoction	Ext., wash	D
		Being used as vitamin source (5)	Fruits	Raw material	O. Ad.	F
		Constipation (3)	Fruits, leaves	Tea, eating of fruits	O. Ad.	
		Cough (2)	Fruits	Tea, eating with honey	O. Ad.	

Rutaceae	*Ruta graveolens* L.	Being used to induce abortion (1)	Leaves, flowers	Tea	O. Ad.	D
		Being used for strengthening of voice strings (1)	Leaves	Chewing	O. Ad.	

Salicaceae	*Populus *sp. L.	Pain (1)	Bud	Ethanol decoction	Ext., compress, rubbing	D
		Disinfection of wounds (1)	Bud	Ethanol decoction	Ext., wash	

Scrophulariaceae	*Verbascum densiflorum* Bertol.	Cough (1), diseases of respiratory tract (1)	Aerial parts	Tea	O. Ad.	D

Scrophulariaceae	*Veronica officinalis* L.	Complicated wounds (1)	Leaves	Compress, compress with honey	Ext.	
		Toothache (1)	Aerial parts	Decoction	Ext., rinsing	

Solanaceae	*Solanum tuberosum* L.	Sinusitis (1), inflammation of the throat (1)	Roots	Compress of boiled roots	Ext.	F
		Cough (1)	Roots	Inhalation	O. Ad.	
		Gastric hyperacidity (4)	Roots	Raw material	O. Ad.	

Tiliaceae	*Tilia cordata* Mill.	Cold, cough, and fever (23)	Flowers	Tea	O. Ad.	D
		Gastric hyperacidity (1)	Bud	Chewing	O. Ad.	
		Diseases of the respiratory system (1)	Flowers	Inhalation	O. Ad.	
			Flowers	Bath	Ext.	

Tropaeolaceae	*Tropaeolum majus* L.	Inflammation of the bladder (2)	Flowers	Tea	O. Ad.	D
		Lungs inflammation (3), cold (2), and fever (2)	Flowers	Tea	O. Ad.	
		Gastric hyperacidity (1)	Flowers	Tea	O. Ad.	

Vibrionaceae	*Viburnum opulus* L.	Bronchitis (2), asthma (3), and cough (1)	Fruits	Eating with sugar, juice with honey	O. Ad.	
		Weak organism (1)	Fruits	Eating with honey	O. Ad.	
		Hypertension (1)	Fruits	Juice with honey	O. Ad.	

## Data Availability

Collected material will be left in Lithuanian Museum of the History of Medicine and Pharmacy.
